# Role of elasticity imaging/B-mode imaging ratio in the evaluation of solid breast lesions

**DOI:** 10.4102/sajr.v29i1.3158

**Published:** 2025-07-23

**Authors:** Asif I. Tamboli, Abhijit A. Gadpalliwar, Raghav Agarwal, Chaitali V. Ukirade

**Affiliations:** 1 Department of Radiodiagnosis, Krishna Institute of Medical Sciences, Krishna Vishwa Vidyapeeth, Karad, India

**Keywords:** breast neoplasm, elasticity imaging techniques, ultrasound elastography, histopathology, EI/BI ratio

## Abstract

**Background:**

Ultrasound elastography, with the measurement of the lesional width ratio between elasticity imaging (EI) and B-mode image (BI) (EI/BI), provides a non-invasive method for breast cancer (BC) characterisation. Evidence from a limited number of researchers supporting the efficacy of this ratio in avoiding unnecessary biopsies warrants further exploration.

**Objectives:**

To assess the role of the EI/BI ratio in the evaluation of solid breast lesions and correlate the findings with histopathological results.

**Method:**

The study enrolled 54 female patients with clinically palpable breast lesions, non-palpable breast lesions (seen on mammography or ultrasound) and high-risk female participants with a positive family history of BC. Using ultrasound elastography, the EI/BI ratio was calculated and correlated with the histology using the Chi-square test and Cramer’s V test.

**Results:**

The mean age was 41.9 ± 11.8 years, and 59.2% had fibroadenomas. The EI/BI ratio was ≥ 1 in 16 (29.6%) cases, where malignancy was confirmed on histology in all cases. Thirty-eight cases were benign as per the EI/BI ratio (< 1), of which 2 were found to be malignant. A significant correlation was seen between the EI/BI ratio and histopathology findings (*p* < 0.001). The specificity, sensitivity, positive and negative predictive values and diagnostic accuracy of the EI/BI ratio were 100%, 88.9%, 94.7%, 100% and 96.3%, respectively.

**Conclusion:**

The EI/BI ratio is effective in differentiating between benign and malignant solid breast lesions, with a statistically significant correlation with histopathology.

**Contribution:**

The study validates the use of EI/BI ratio by radiologists to effectively differentiate between benign and malignant breast lesions in patients.

## Introduction

Breast cancer (BC) is the most common malignancy among women with an annual global incidence of 2.1 million, causing the highest number of cancer-related deaths in women.^1^ It is also the most predominant cancer among Indian female participants with prevalence and mortality rates as high as 25.8 and 12.7 per 100 000 women, respectively.^2,3^ Hence, screening and early detection are important for improving outcomes and survival in such patients.^1^

Mammography and ultrasound are the commonly employed diagnostic modalities for BC because of their high sensitivity.^4^ Nontheless, there is a likelihood that mammography may yield false-negative results when performed on dense breasts and ultrasound lacks specificity because solid lesions may be benign.^5^ To overcome this, the Breast Imaging-Reporting and Data System (BIRADS) was introduced by the American College of Radiology. However, the BIRADS criteria may also generate false positive results leading to unnecessary biopsies.^6,7^

A more accurate, non-invasive method used for BC evaluation is ultrasound elastography.^4^ It assesses the relative tissue stiffness by measuring the displacement (strain) in response to a mechanically applied force.^8^ A real-time analysis of the returning radiofrequency signals is acquired using the standard B-mode image (BI) algorithm and the compression elasticity imaging (EI) algorithm.^9^ The breast is the only organ where tumour size differs between BI and EI, with malignant lesions appearing larger in the latter, because of the invasive nature of BC.^10^ This allows diagnostic characterisation by measuring the ratio of the maximum diameter of the lesion on EI to that on BI (EI/BI = width ratio).^11^ This ratio has shown high sensitivity (99%) and specificity (87%) for values > 1 suggesting malignancy and < 1 implying benignity.^12^ The EI/BI ratio has also shown significant correlation with tumour grades.^10^

There is limited literary evidence that explores the use of ultrasound elastography in the potential diagnosis of BC. This research is therefore aimed at assessing the role of the EI/BI ratio in the evaluation of solid breast lesions and correlation of the findings with histopathological results in a tertiary care hospital in Karad, India.

## Research methods and design

### Study design and participants

A prospective, observational, analytical study was conducted at a tertiary care hospital in India, between 01 June 2024 and 31 December 2024. Female patients with clinically palpable and non-palpable breast lesions (seen on mammography or ultrasound) and high-risk female participants with a positive family history of BC, who were scheduled for breast ultrasound, were included after obtaining written informed consent. Patients with cystic breast lesions, recurrent BC following chemotherapy or radiotherapy, and pregnant women, were excluded.

The sample size was calculated using Buderer’s formula (Equation 1)^13^:


$$n=(Z_{1-\alpha /2}^{2}\times S_{\text{N}}\times [1-S_{\text{N}}])/(L^{2}\times \text{Prevalence})$$ [Eqn 1]

where, *n* = required sample size, *S*_N_ = anticipated sensitivity, α = size of the critical region (1 – α is the confidence level), *z*_1−α/2_ = standard normal deviate corresponding to the specified size of the critical region (α), and *L* = absolute precision desired on either side (half-width of the confidence interval) of sensitivity. The prevalence was considered to be 20%, a margin of error of 10%, and a 95% confidence level (*Z* = 1.96). The prevalence data in a study by Farooq et al. closely matched the demographic and clinical characteristics of our target population. They found that the sensitivity of elastography for differentiating benign from malignant breast lesions was 92%.^14^ We used 92% as a value for the sensitivity. Based on these parameters, the required sample size was approximately 61 participants, with an adjustment for a 10% non-response rate suggesting a final target of approximately 68 participants. Because of practical constraints, 54 participants were ultimately enrolled and included in the analysis.

### Ultrasound procedure

Detailed clinical history and physical examination findings were recorded from each patient. Ultrasound elastography was performed using an ACUSON S2000 diagnostic ultrasound system (Siemens Medical Solutions, CA, US) with a 4 MHz – 6 MHz, 9L4 linear probe. The BI of the lesion was determined initially, followed by the EI, acquired using Acoustic Radiation Force Impulse (ARFI) technology and Virtual Touch Imaging (VTI). The BIRADS staging was determined as well.

The lesion width was measured in the same location on the BI and EI. The EI/BI ratio (width ratio) was then calculated by dividing the maximal horizontal length of the lesion measured on the EI by the corresponding length measured on the BI. If an echogenic ring was present around the lesion on BI, it was not included in the measurement. An EI/BI ratio ≥ 1 was considered as malignant while < 1 as benign.^10,12^ Histopathological examination was conducted on biopsied samples from these lesions. The EI/BI ratio was correlated with the histopathology findings, extracted from the patient’s medical records.

### Statistical analysis

Data were compiled and analysed using Microsoft Excel and statistical software R version 3.6.3. Continuous variables were presented as mean ± standard deviation (s.d.) while categorical variables were presented as number (%). The Chi-square test was used to evaluate the association between attributes; a *p* value of *≤* 0.05 was considered statistically significant. Strength of association was measured by Cramer’s V/odds ratio.

### Ethical considerations

The study was conducted in accordance with the Declaration of Helsinki. All patients provided signed written informed consent before being enrolled in the study. The study protocol was approved by the Institutional Ethics Committee of the University (reference number: KVV/IEC/07/2024). The data collected were anonymised to ensure patient privacy.

## Results

A total of 54 female patients were included in the study. The number of patients aged between 40 and 49 years was higher than other age groups (37.0%), and the mean age was 42.0 ± 11.8 years. The right breast was involved in 72.2% of cases. Table 1 presents a summary of the various parameters analysed in the study. Of the various breast tumours, fibroadenoma was diagnosed in 59.3% of the patients. The BI revealed that 72.2% of the lesions were well-circumscribed and 48.1% were lobulated. The EI/BI ratio was ≥ 1 in 16 (29.6%) cases, where malignant transformation was confirmed with ultrasound elastography and histopathology. The remaining 38 cases were found to be benign by EI/BI ratio (< 1), of which 2 were found to be malignant on histopathology.

**TABLE 1:** Baseline characteristics of patients included in the study.

Parameter	Subcategory	*n*	%
Age (years)	20–29	9	16.7
	30–39	12	22.2
	40–49	20	37.0
	50–59	11	20.4
	60–69	1	1.9
	70–79	1	1.9
Breast involved	Left	15	27.8
	Right	39	72.2
EI:BI ratio	< 1	38	70.4
	≥ 1	16	29.6
BIRADS staging	B II	20	37.0
	B III	18	33.3
	B IV	12	22.2
	B V	4	7.4
Features of lesion on B-mode image	Lobulated	26	48.2
	Spiculated	11	16.7
	Well-defined	19	35.2
Lesion margin on B-mode image	Well-circumscribed	39	72.2
	Irregular	15	27.8
Ultrasound elastography finding	Benign	38	70.4
	Malignant	16	29.6
Tumour type (histopathological finding)	Fibroadenolipoma	4	7.4
	Fibroadenoma	32	59.3
	Ductal carcinoma *in situ*	11	20.4
	Invasive ductal carcinoma	7	13.0

BIRADS, breast imaging-reporting and data system; EI:BI, elastography image: B-mode image.

The Chi-square test showed no significant correlation between the breast involved and the histopathology findings (*p* = 1.00). However, a significant correlation was found between the EI/BI ratio and histopathology findings (*p* < 0.001). From Cramer’s V (0.9177), the strength of correlation was found to be very high (Table 2).

**TABLE 2:** Correlation between histopathology findings, breast involved and elastography image: B-mode image ratio.

Parameter	Sub-category	Histopathology findings		Test	
		Benign	Malignant	Chi square *p*-value	Cramer’s V
Breast lesions	Left	10	5	1.000	-
	Right	26	13	-	-
EI:BI ratio	< 1	36	2	< 0.001*	0.9177
	≥ 1	0	16	-	-

EI:BI, elastography image: B-mode image.

* , *p* ≤ 0.05 is considered significant.

**FIGURE 1:**
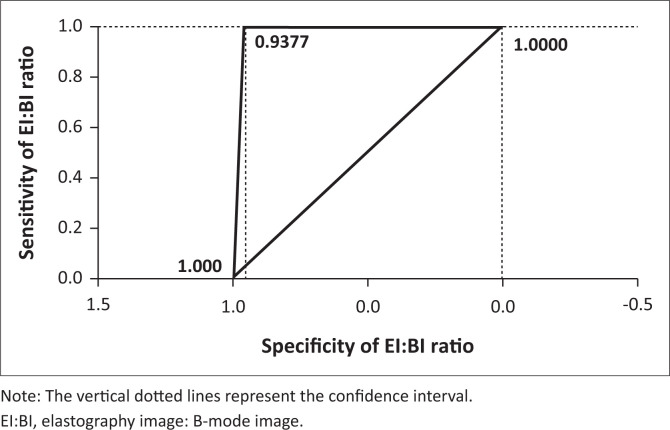
Receiver operating curve of sensitivity and specificity in evaluating solid breast lesions.

Figure 1 depicts the receiver operating curve (ROC) of sensitivity and specificity. The area under the ROC (AUROC) was observed to be 0.9737 (0.9377–1.0000). The sensitivity, specificity, positive predictive value (PPV), negative predictive value (NPV) and diagnostic accuracy of the EI/BI ratio for classifying breast lesions into benign and malignant were found to be 100%, 88.9%, 94.7%, 100% and 96.3%, respectively (elastography correctly diagnosed 52 of the 54 lesions).

## Discussion

This study assessed the role of the EI/BI ratio in the evaluation of solid breast lesions and correlates the findings with histopathological results. While all lesions with EI/BI ≥ 1 were indeed found to be malignant on histopathology, 2 of the 38 lesions with EI/BI < 1 were subsequently diagnosed as malignant on biopsy. However, the ultrasound elastography – histopathology correlation was found to be statistically significant.

The results closely mirror the observations by Barr et al. and Destounis et al., who measured the largest dimensions of the EI and the BI images, represented by the central hypoechoic part of the lesion and excluding the echogenic halo.^12,15^ They concluded that the EI/BI ratio of ≥ 1 indicated maliganancy.^12,15^ They also noticed that ultrasound elastography and the EI/BI ratio were associated with high sensitivity in characterising benign and malignant lesions of the breast.

Similarly, Destounis et al. also observed that in 100 malignant lesions, 99 had an EI/BI ratio ≥ 1, while 1 had EI/BI ratio < 1. These results indicated 99% sensitivity, 91.5% specificity, 90% PPV and 99.2% NPV, all of which are highly comparable to the current research.^15^ Using the same cutoff value of 1 in a large multi-centre trial evaluating 635 biopsy-proven cases, Barr et al. observed a sensitivity of 99% and specificity of 87%.^12^ Alhabshi et al. used 1.1 as the cutoff point for width ratio and found a specificity of 84% in detecting breast malignancy, suggesting that it could reduce the need for unnecessary biopsy for benign lesions with indeterminate or equivocal features.^16^

In concordance with this study, Grajo et al. found a significantly positive correlation between the width ratio and tumour grade (*p* < 0.001).^10^ Because histologic grading is a critical determinant of cancer prognosis, this could potentially advocate for exploring the EI/BI ratio cutoff values for the different tumour grades.^10^ Moreover, an increased width ratio, despite relatively less conspicuous malignant ultrasound characteristics (such as posterior acoustic enhancement, microlobulated margins, smaller mass size, inconspicuous margins) could raise the suspicion of high grade BC. This could guide a more vigilant surveillance for satellite lesions or axillary adenopathy, subsequently influencing staging, prognosis, and management of these cancers.^10,17^

This study confirms and supports that ultrasound elastography along with routine BI, provides a convenient, practical, and non-invasive method for characterising breast lesions as benign or malignant. This may potentially reduce the number of unnecessary biopsies and invasive procedures, particularly for BI-RADS III or IVa lesions.

### Study limitations

This was a single-centre study with a small sample size, and the outcomes cannot be generalised to a broader population. In breast ultrasound, high-frequency linear probes, typically operating in the 10 MHz – 15 MHz range or higher, are routinely utilised to optimise spatial resolution and tissue detail, both of which are critical for accurate lesion characterisation. In this study, a 4 MHz – 6 MHz (9L4) linear probe was used. The choice of a lower-frequency probe may be justified in cases requiring greater depth penetration, such as imaging in patients with large breasts, deeply located lesions or certain body habitus considerations. However, it is important to recognise that the use of a lower-frequency probe may result in decreased image resolution, particularly in the superficial tissues where many breast lesions are found. This could potentially impact the visualisation of lesion margins, internal architecture and subtle tissue abnormalities, thereby affecting diagnostic accuracy and BI-RADS categorisation. As such, the use of the 4 MHz – 6 MHz probe represents a methodological limitation of this study and should be taken into account when interpreting the results. Another limitation of this study is the lack of stratification of the EI/BI ratio findings against tumour grade or BI-RADS categories because of sample limitations. While the overall relationship between EI/BI ratios and lesions was explored, stratifying these findings according to tumour grade and BI-RADS categories would have provided a more granular understanding of their diagnostic value. Such stratification could potentially reveal how EI/BI ratios vary across different tumour grades (e.g. low-grade versus high-grade) and BI-RADS categories, further refining their role in tumour characterisation and malignancy risk assessment. Nonetheless, this study provides important data that support the effectiveness of ultrasound elastography in the diagnosis of BC.

## Conclusion

This study validates the use of the EI/BI ratio for the evaluation of solid breast lesions, demonstrating a statistically significant correlation between the ultrasound elastography and histopathology. Ultrasound elastography can be used in conjunction with other imaging modalities to effectively diagnose and differentiate between the malignant and the benign lesions. Large multicentric, long-term, prospective studies with larger sample sizes are needed to validate the results and establish the utility of ultrasound elastography for the diagnosis of BC.
